# Editorial: Remote sensing for field-based crop phenotyping

**DOI:** 10.3389/fpls.2024.1368694

**Published:** 2024-01-26

**Authors:** Jiangang Liu, Zhenjiang Zhou, Bo Li

**Affiliations:** ^1^ State Key Laboratory of Vegetable Biobreeding, Institute of Vegetables and Flowers, Chinese Academy of Agricultural Sciences, Beijing, China; ^2^ College of Biosystems Engineering and Food Science, Zhejiang University, Hangzhou, Zhejiang, China; ^3^ Syngenta, Jealott’s Hill International Research Centre, Bracknell, United Kingdom

**Keywords:** smart agriculture, remote sensing, crop phenotyping, computer vision, machine learning, data fusion

With the population predicted to increase to over 9.6 billion by 2050, and food demand anticipated to increase by between 60 and 100%, sustainable and resilient agricultural production with a minimised impact on the environment is crucial particularly at the context of global climate change. Breeding and identifying crop varieties with high production and adopted specific environmental conditions requires considerable efforts to assess crop phenotypic traits (e.g., LAI, plant height, biomass, yield et al.), which contribute to the stable increased productivity and efficient use of resources. Traditional methods for determining crop phenotypic traits are mainly based on destructive field sampling, hand-held instrument measurement, which are characterized as time-consuming, limited representative. Remote sensing provides a novel solution to quantify crop structural and functional traits in a timely, rapid, non-invasive, and efficient manner for field crops. With the development of sensors and diversified algorithms, a range of crop phenotypic traits have been determined in a manner of high-throughput, including morphological parameters, spectral and textural characteristics, physiological traits, and responses to abiotic/biotic stresses under different environments.

This Research Topic presents 15 research articles and 2 reviews with an insight into the recent advances in crop phenotyping using remote sensing to address some high-priority challenges including comparing ground-based handheld and remote aerial system, plant biophysical parameters estimation with innovative traits, imagery data fusion and investigating the impact of experimental design on the performance of traits extraction.

The current state of art high-throughput phenotyping platform including ground-based and aerial platforms. The integrated imaging sensors such as visible light, 3D, hyperspectral and fluorescence sensors were reviewed by Cudjoe et al., and pointed out that the lack of appropriate field phenotyping infrastructures is impeding the development of new crop cultivars with improved traits and will eventually have a negative impact on the agricultural sector and African food security. Due to the operational complexity and limited funding, the deployment of efficient and low-cost high-throughput phenotyping methods are still highly demanded in Africa. In addition to ground-based and aerial-based remote sensing, Lin et al. also described satellite-based remote sensing in potato yield prediction. Furthermore, strategies for potato yield prediction including remote sensing, crop growth models (CGM) and yield limiting factors were discussed in depth, and the application of different CGMs were analysed. As data from solely single sensor source often limits the performance of prediction model, Lin et al. proposed that multi-source data fusion and time-series data have enormous potential for future potato yield prediction. Marzougui et al. investigated two data fusion methods for unmanned aerial systems (UAS) multispectral imagery and high-resolution satellite imagery in field pea yield prediction, showing the improved model performance by fusing multiple time point and multiple sensor source information. With using only UAS, Liu et al. implemented feature fusion between texture features extracted from RGB imagery and spectral features from multispectral imagery for estimating the frost damage index in lettuce. Bai et al. applied more comprehensive sensor data fusion in an existing Field-based High-Throughput Plant Phenotyping (FHTPP) system and combined morphological, spectral, thermal, and environmental features as the inputs of the machine learning models to estimate the cover crop biomass. Shi et al. recognised the limitation of using single-source remote sensing spectral or LiDAR waveform data for Leaf Area Index (LAI) estimation and presented another example showing the advantage of data fusion. Rather than using empirical model that always requires sufficient amount of field measurements and does not provide a good explanation of the fusion mechanism, physical model geometric-optical and radiative transfer (GORT) integrating both spectral imagery and LiDAR waveform showed an enhancement in LAI estimation comparing with using spectral or LiDAR data alone. As a critical physiological and biochemical parameter indicating crop growth status and yield potential, LAI estimation was also investigated for winter wheat and maize in another two studies by Zou et al. and Sun et al. Although both studies use single multispectral imaging sensor equipped on UAS, both spectral and texture features were extracted and fused as the input variables of machine learning model. Multiple multivariate statistical regression models were constructed and compared, and all presented high performances. All the texture features mentioned above are based on the grey level co-occurrence matrix (GLCM) method, however, window size and direction texture parameters are highly sensitive to texture metrics and default parameter values are always applied in previous studies. As a result, Liu et al. conducted a detailed study to understand the optimum window size and directional parameters at different growing stages of rice. As most of the prediction models were developed for a specific growth stage in previous studies, Pokhrel et al. integrated growing degree days (GDD) with VIs extracted from UAV-based multispectral imagery for estimating intercepted photosynthetically active radiation (IPARf), radiation use efficiency (RUE) and harvest index (HI), which were three yield-contributing physiological parameters of cotton. The incorporation of GDD allowed the prediction to be reliably made throughout the whole growing season.

Many VIs were developed in the past decades showing correlation with crop physiological parameters, however, none of the previous studies investigated VIs that can be applied to predict the corn yield throughout the season. Shrestha et al. applied both correlation analysis and random forest model to identify the VIs with the most consistency and highest predictive power for corn yield prediction.

Crop traits evaluation on a row segment basis within plots is common in field phenotyping. Tolley et al. conducted UAS flights and extracted crop traits using RGB, LiDAR and VNIR (visible and near infrared) sensors and concluded that significant difference was observed between different row selections, and with large plot size, excluding outer rows could lead to more robust model performance. It is believed that this study can support long-standing principles of experiment design in both agronomy and crop breeding with remote sensing.

Apart from aerial platform, ground-based system is also popular in crop phenotyping due to the high resolution imaging across a wide range of wavelengths. Tuerxun et al. used a portable spectroradiometer to obtain the hyperspectral data, and multivariate regression model was developed for chlorophyll content estimation in jujube leaves after feature reduction. He et al. applied a handheld spectrometer to generate VIs and a light quantum sensor to measure photosynthetic active radiation, which showed fairly good relationships with the dry mater of Choy Sum. Although the same VIs can be derived from both ground-based and aerial-based systems, it was interesting that Herr and Carter found poor correlation across remote sensing platforms, which suggested that data collected from different systems should not be used interchangeable.

Crop phenotyping not only can assess the crop growth status, but also support the decision of the crop management strategy. Deng et al. collected phenotypic data to support the conclusion that the umbrella-shaped trellis system largely improved the production of Donghong Kiwifruit while maintaining the fruit quality.

Zhou et al. presented the only research of ground-based 3D model reconstruction and analysis in this topic. New algorithm was proposed for skeleton point search, which facilitated the stem and leaf segmentation, further leading to the five phenotypic parameters estimation including plant height, stem diameter, main stem length, regional leaf length and leaf number.

The studies published in this Research Topic present novel computer vision algorithms and provide new knowledge in the broad field of crop phenotyping. It is obvious that UAS integrating with imaging sensors has shown great advantages in crop phenotyping. Future studies should investigate more on multi-sensor data fusion and multiple time point data fusion for more reliable crop traits estimation under various environmental conditions. As data collected from different remote sensing systems cannot be used interchangeable, standardisation of remote sensing pipeline for crop traits estimation needs to be investigated in the future to provide re-usable data and reduced operational cost. We hope the studies presented in this Research Topic will help consolidate the integration between remote sensing and crop phenotyping, resulting in more reliable and affordable phenotyping tools under dynamic environments.

## Author contributions

JL: Conceptualization, Funding acquisition, Writing – original draft, Writing – review & editing. ZZ: Writing – review & editing. BL: Conceptualization, Resources, Writing – original draft, Writing – review & editing.

